# The Care of the Insane in Texas*This address was delivered by Dr. M. L. Graves, Superintendent of the Southwestern Insane Asylum, San Antonio, Texas, to the faculty and students of the Medical Department of the University of Texas, and the general public at Galveston, Friday night, March 24th, by invitation of the faculty of the University.

**Published:** 1905-04

**Authors:** M. L. Graves


### TEXAS MEDICAL JOURNAL.

ESTABLISHED JULY, 1885.

PUBLISHED MONTHLY.—SUBSCRIPTION $1.00 A YEAR.

VOL. XX. AUSTIN, APRIL, 1905. No. 10.

For Texas Medical Journal.

### The Care of the Insane in Texas.*

BY M. L. GRAVES, M. D.

Fully approving the purpose of this series of lectures for public information, and cordially appreciating the honor done me by your excellent faculty in asking me to deliver the address upon "The Care of the Insane," I come to you tonight with a message and an appeal—a message from the imprisoned victims of disease, who pine away days of sunshine with lamentations of sorrow, who need and who deserve every consideration at the hands of their more fortunate fellow-beings, and every assistance, legislative and otherwise, that will contribute to their cure or their comfort.

I come with an appeal from hundreds of helpless human beings now confined in jails, poorhouses and private homes of Texas; and yet other hundreds of homes and relatives struggling under a burden of sorrow well nigh intolerable, and embarrassed by a responsibility they are unprepared to meet.

It is quite appropriate that this message be delivered to students of science who gather here to learn the Ars Medicatrix and whose professional future must bring them into close contact with the unsound of mind as well as the diseased of body. You will see them in the very incipiency of the mental malady, when prompt recognition and proper treatment may achieve most noteworthy re-

*This address was delivered by Dr. M. L. Graves, Superintendent of the Southwestern Insane Asylum, San Antonio, Texas, to the faculty and students of the Medical Department of the University of Texas, and the general public at Galveston, Friday night, March 24th, by invitation of the faculty of the University.

sults. You are thus interested because you may be called upon to diagnose the case before public attention has become enlisted, but not before public safety becomes endangered. You are interested from a sociological point of view, because the doctor is in a sense the guardian of society and responsible for the integrity of his race. By your wise advice and counsel you can subserve the ends of society by many means tending to prevent the pollution of its blood-current.

He who thinks the proper care and treatment of this constantly augmenting population is an easy problem, and one already solved, does not understand the real condition. It is engaging today the serious thought of our humanest men, and should receive the practical consideration of our philanthropists who seem to be seeking some useful way in which to dispose of their surplus wealth.

In some States a large amount of the current operating expenses of the insane hospitals is met by a fixed income from railroad bonds, industrial stocks, and other securities which have been donated to the institution by the benevolent and beneficent rich; indeed, in a number of instances, large buildings have been erected by private donations, but so far as I know, and am advised, no Texas philanthropist has sought our Texas hospitals for the insane for the dissipation of his plethoric bank account.

I may say, however, without infringement upon the rights of our sister institutions, that the San Antonio asylum stands ready to welcome the first invasion of this field of philanthropy upon the part of our wealthy and humanitarian citizenship.

Texas is a magnificent State, imperial her domain, incalculable her resources, splendid her citizenship. She assumes the care and treatment of 3500 disordered minds in a humane and generous spirit. I shall discuss this subject under three heads:

### FIRST—OUR INSTITUTIONS.

We have three hospitals for the insane proper, and one colony for epileptics.

The first institution established in Texas was the State lunatic asylum at Austin. It is located nearest the geographical center of the State, and was opened in 1861. It remained our only institution for the insane till 1882, when the pressure for room made legislative appropriation necessary, and the North Texas hospital was located at Terrell, in the black-land belt. It was opened in 1885, and was immediately filled to its fullest capacity.

It soon developed that these two institutions were incapable of accommodating the ever augmenting army of insane. Accordingly in 1890 legislative provision was made for a small institution at San Antonio, where climatic conditions were more favorable and where large sections of territory were immediately accessible. Opening in 1892, the institution had a maximum capacity of 275 patients, until 1899, when additional buildings became available, increasing the capacity to 720 patients, and this additional room was promptly taken, and has been taxed to the utmost ever since.

Owing to the repeated and urgent reports of the asylum authorities recommending a separation of the epileptics and the increasing number of epileptics, the Legislature made an appropriation of $200,000 in 1899 for the erection of a colony at Abilene, and this institution was opened on the 26th day of March, 1904, with 104 epileptics taken from the previously named institutions.

At the close of the first fiscal year of the State lunatic asylum at Austin in 1861, there were about fifty patients.

At the close of the fiscal year ending August 31, 1904, there were confined in all the Texas institutions a grand total of 3487 insane and epileptic patients. This is an increase of 6800 per cent.

| | |
|---|---|
| General population of Texas, U. S. census 1860.... | 604,000 |
| Insane population in Austin 1860, about.......... | 50 |
| Ratio of insane to sane population, 1860.......... | 1 to 12,080 |
| General population of Texas, U. S. census, 1880... | 1,596,749 |
| Insane population in asylum, 1880............... | 369 |
| Ratio of insane to sane population, 1880.......... | 1 to 4,313 |
| General population of Texas, U. S. census 1900.... | 3,048,710 |
| Insane population asylums, 1900................ | 2,379 |
| Ratio insane to sane population, 1900............ | 1 to 1,281 |
| Insane population in all asylums, 1904........... | 3,487 |
| Ratio insane to sane population, 1904............ | 1 to 874 |
| Increase of general population, Texas U. S. census, in last 45 years ........................... | 504 per cent |
| Increase insane population asylums in last 45 years. | 6800 per cent |

The management of all these institutions is practically identical, and, strange to say, there has been little material change in the law governing the trial and commitment of lunatics since the Act of February 5, 1858.

Under our present statutes the management and control of all asylums heretofore established, and any others that may be hereafter established, vests in a board of managers, subject to such rules and regulations as may be prescribed by legislation.

It should be stated in this connection that no money of all the large sums appropriated for the support and maintenance of our institutions is kept on hand at the institutions; every dollar is kept in the State Treasury, and paid out only on approved or certified bills, audited and found correct, and passing through appropriate channels.

None of the managers, officers or employes are in any way connected with the sale of merchandise, supplies or other articles to the asylum, nor do any of them have any interest in any contract therewith.

All sums of money received at the asylum are immediately forwarded to the State Treasurer by the Superintendent, so that the institution handles no funds, and its main officers are under bond of $10,000 each, and it is difficult to see how a more perfect system for the protection of public funds in our institutional management can be devised than that existing under the laws of Texas.

It is gratifying to say in this connection, that our State Government has been singularly free from corruption and scandal in management, and this may be confidently stated to be due to the wise provision of the Legislature in statutory enactment, to constant executive oversight and supervision, as well as to the integrity of the officials selected.

### SECOND—OUR LUNACY LAWS.

If it be true that the laws of a nation or a people are an index of their civilization, Texas must admit with some degree of humiliation that she is not yet in the forefront of the world's great march of progress, for we cling to ancient and obsolete practices; we maintain a custom and follow a law designed when lunacy was regarded as a crime, and not a disease. While our laws have been amended from time to time, the essence and substance have remained the same for the last half century. We still follow criminal methods of procedure in the apprehension, trial, and commitment of our insane to the hospitals. In fact, the legal name of every institution in this State is asylum or insane hospital.

First. Let us discuss the apprehension and trial of an insane patient:

Article 128 of the Revised Statutes states: If information in writing and under oath be given to any county judge that any person in his county is a lunatic, or *non compos mentis*, and that the welfare of himself or others require that he be placed under restraint, and said county judge shall believe such information to be true, he shall forthwith issue *warrant* for the apprehension of such person, and shall fix a day for hearing, and determination of the matter.

How like a criminal procedure throughout! What justification can be made for such a course? A warrant directed to a sheriff who goes armed with six-shooter and handcuffs, as if they were necessary, and who seeks to arrest the victim of disease—reads the warrant, arrests and drags him, indignant and perhaps resistant, to the county jail, and there incarcerates him with felons of every description until the day he is brought into court, dazed and disgusted, timid and cowering, or indignant at his treatment, and before a jury, is actually prosecuted by an attorney-at-law for *being sick in mind;* and he is compelled to sit and listen to all testimony of his conduct, and hear a jury file in and pass sentence upon him, and then he is returned to jail, awaiting his transfer by the same sheriff armed with pistol and handcuffs, perhaps, to an institution which purports to be a hospital and *treat the sick.* What an absurdity! What an outrage! What would the patients of John Seely hospital, and what would the people of the State think if it were necessary to empanel a jury and try all your patients for typhoid fever, and pneumonia, and pleurisy, and direct a sheriff to take them to a hospital for care and treatment after the jury had passed upon the condition of the patient, and a verdict of guilty had been rendered?

It is true, the regular session of 1903 amended Articles 128 and 129 regarding the apprehension of lunatics so that trials can now be held at the home of the accused "to prevent the deleterious effect which such arrests and removal for trial has upon the disordered mind."

But why any *trial* at all? Why any jury? Why any sheriff? Why any court scene at the home or at the court house? It is all unnecessarily cumbersome, expensive, unjust and offensive to any sensitive patient. It neither safeguards the rights of the patients nor protects the public. It is out of date and has been abolished by our most progressive States. Moreover, the trial is not used in private cases—and why this class distinction? If protection of person or property is necessary, certainly the public trial of private patients is as necessary as for public patients. Recognizing insanity as a disease—let the committal of its sufferers to our public hospitals be done by the certification of competent medical men, who examine the patient professionally, and make the proper affidavits before the county judge, who may summon any other evidence thought necessary, and let the patient be ordered to a hospital in this humane and effective way without public trial in any way.

Exceptions could be made of criminal cases and of all those where property rights are endangered, or serious family difficulties may occur, or where contentious persons demand a trial—in which event the law could be made optional, not mandatory—but in my judgment *criminals* should always have the public trial.

In the foregoing recital of the law governing admission of our institutions nothing was said of criminal cases; that is, persons charged with crime who enter a plea of insanity as a defense. It may be said with a feeling of pride, that the spirit of our people universally revolts at the plea of insanity as a defense in any of the major crimes, and not unusually a strong prejudice exists as to any such claim and people immediately jump to the conclusion that this plea is invariably an "insanity dodge," and should receive scant consideration. Let me say in this connection, that I myself have no more sympathy with the "insanity dodge" than any man within the confines of this State; but an experience of more than six yaers in the constant study and care of the insane, and an observation of court processes in not a few instances of this character, lead me to believe this danger greatly overestimated, and escape from penalty of the law by impostors much less than generally believed. Indeed, I must say, in this connection, *a homicide or other felony well planned or deliberate may be no more evidence of sanity than insanity.*

When a crime has been commited by a person who is actually diseased of mind, it is wrong to punish such person as a criminal, and every fair-minded man should be willing to give this plea fair consideration; but the public safety should be safeguarded at all times. An experience of six years among the insane, and to a certain extent in the criminal courts, has only given me an observation of two cases where the plea of insanity was a distinct dodge, and in neither of these cases did the criminal escape the just punishment of the law. It must be said in this connection, also, that more men who are actually insane commit crime under the dominance of an insane delusion and are promptly convicted with never the thought of a plea of insanity as a defense, and who actually serve their sentences, than there are of men who escape just punishment, upon the impostor's plea of insanity.

Some time since I had an application from the superintendent of the penitentiaries at Huntsville, asking for the admission of twenty-six lunatics, who were serving sentences in the penitentiaries. A large number of these were for homicide, rape, and major crimes. Without any knowledge of their history, I hazard the opinion that a goodly proportion of these were actually insane at the time of the commission of the crime for which they were serving sentence, and that no one thought of a plea of insanity as a defense.

Perhaps I should give you the mode of procedure in criminal cases. Under existing conditions, a person is charged with a crime, and upon trial of the case in a district court, enters a plea of insanity as a defense. Two courses are opened: one is to permit trial to proceed, and the jury to take the evidence of insanity in connection with other evidence introduced in the case, and finally render a verdict in accordance therewith; under these conditions the jury may find defendant guilty, and assess punishment at death or confinement in the penitentiary, without giving due heed to testimony as to insanity, and he may actually pay the penalty for his criminal conduct, although he may be insane at the time of the commission of the deed, at the time of the trial, and at the time of the execution of the penalty. On the contrary, he may be acquitted of the charge and turned loose upon the community, an insane man; subsequently, perhaps, to find his way into the asylum through the ordinary county court process.

Another proceeding is as follows: During a trial, when a defendant enters a plea of insanity, the district judge immediately stays the criminal trial, empanels a jury, and tries the defendant as to mental soundness. If he be declared to be of unsound mind, the court immediately orders his commitment to an insane hospital, and defers the trial for his crime until the patient is discharged by the hospital authorities as cured, in which event he must be returned to the sheriff to stand trial for his crime. If he be acquitted of the charge of insanity during the district court trial, the criminal court trial still goes on.

It is very rare for courts to inflict penalty upon those criminals adjudged insane. In the district court, where criminal trial has been stayed and the criminal committed to an institution, upon his return from the asylum he is usually acquitted at the time, on the ground that he was insane when the deed was committed.

Another way in which criminals may seek to escape the ends of justice is as follows: A crime is committed most likely in some other county than county of defendant's residence; he is indicted by the grand jury and bound over to the district court; he secures bond and gets out of jail, returning to the county of his residence among relatives and friends, and there after some manifestation or peculiar conduct, or violent outbreak, is charged by his relatives with insanity, taken before the county court, promptly adjudged insane without a dissenting voice, and with no knowledge of the crime committed in the other county before the court.

He is promptly conveyed to the insane asylum, remaining for an indefinite period of time, most likely till after the first trial is called, when postponement is entered on account of defendant's incarceration in the asylum. The authorities of the asylum may in the meantime have no knowledge whatsoever of the criminal charge, and their testimony may be sought under these conditions to defeat the ends of justice.

It must be said, however, in this connection, that the large majority of criminal trials in this State, where insanity is entered as the plea of defense, the physicians of the asylum are never called upon to examine and pronounce upon the sanity of the person so accused.

It should be stated that three classes of cases give us particular trouble:

First—Those having property — the inheritance or disposal of which may be in doubt or in danger—and where covetous or contentious relatives precipitate and perpetuate trouble.

Second—Those in which family disagreement exists as to mental unsoundness or necessity for restraint. This is more directly the case when husbands or wives are incarcerated and family differences may not only jeopardize the peace and cure of the patient, but also the life of the hospital authorities made very unpleasant.

Third—The criminals whose relatives and friends not infrequently desire their release shortly after the criminal case has been settled or sufficient time has elapsed to weaken the criminal liability, and probably escape any punishment.

This is also true of general cases of insanity among those who have committed crimes, and who may improve or recover under asylum treatment. They have usually been incarcerated a long time in jail and become quite reluctant to remain longer deprived of liberty.

It requires no small degree of stamina to resist the appeal and demand for freedom in this class, where a proper regard for public safety is had, and it must be admitted an indisposition of the asylum superintendent, to be considered a party to the so-called "insanity dodge."

Owing to the public discussion of such cases from time to time, I desire on this occasion to make plain my views in criminal cases.

It is my deliberate conviction that all insane criminals and criminal insane who plead insanity as a defense for such major crimes, as homicide, rape, etc., upon due conviction of insanity, should be committed to an insane hospital by our district courts for not less than five years, and then released only upon the certificate in writing of the hospital authorities, attesting the full recovery of the patient, and recommending his discharge, to be filed with the Governor of the State, and to receive his approval, before the patient is released.

A better plan still would be a written certificate from a commission composed of all the asylum superintendents of the State, who shall familiarize themselves with the case, and finally, upon the recommendation of the superintendent in whose care the criminal may be, make a final examination, and certify in writing to the Governor as above.

In my opinion, their judgment should be unanimous, and their recommendation without any restriction filed with the Governor.

This course will fully protect public safety, and will thoroughly safeguard the interest of the patient and can not be reasonably objected to by all genuine cases, and it will prevent the fraudulent ones from seeking to evade the law by a plea of insanity in all the minor criminal cases, and in the major ones it will present so many obstacles to success and so much certainty of failure to the impostor, that it will be rarely sought, and it will be safe to add that very few criminals will escape the just penalty of the law under such a system. Moreover, it is a growing conviction that the criminal insane and insane criminal should no longer be permitted to occupy institutions with the ordinary insane who have no criminal habits or instincts. It is unjust to the latter, demoralizing to their good order, and destructive to their peace and enjoyment; furthermore, it requires stronger restraint, and more secure provision, and much greater restriction of liberty and personal privileges.

A few of the criminal cases will restrict the liberty and interfere with good order and discipline of an entire ward or whole institution.

It is my belief that an institution will subsequently have to be established for this class of cases, and in the meantime a separate and stronger pavilion with a large force of employes and greater security in every way can be erected at one of our present institutions and all insane criminals from the penitentiaries and jails of this State, and all the insane who develop criminal and dangerous habits in the insane hospitals should be confined therein.

I believe provision should be made at Abilene to accommodate all epileptics of every class and character, and they should not be longer tolerated in any asylum proper. With the segregation of the criminal and epileptics, and the isolation of the tuberculous, our hospitals for the insane would take on a larger liberty, and more beneficent results would be immediately appreciable.

THIRD—THE MENACE OF INSANITY.

First—Heredity. No problem of sociology is higher, wider or deeper in significance and in importance to the individual and the race than heredity. It knocks alike at the hovel and the palace gate—it spreads its blessings and its curses on every race, and in every country; it counts its victims by the million and scatters ruin and degradation in countless homes—it breeds anarchy as well as declares law—it promotes poverty oftener than it produces plenty, it spreads disease more widely than it engenders health, it undermines body, destroys mind, and pollutes the very blood-current of society; it is a deadly breath of deadly miasma upon the body politic as well as the body social.

It is steadily and surely multiplying the seeds of evil in the human race; it wrecks character, destroys home, and leaves a trail of suffering and despair, yet how few spend upon it the poor homage of a passing thought, and still fewer heed its dreadful lesson! Surely and steadily it will force itself upon human attention.

We spend much time in breeding our live stock; pedigrees are sought and keenly scrutinized, and enormous sums of money paid for pure breed in animals. But what of our children? Those unfit by reason of mental, physical and moral degeneracy are still permitted to marry without restraint or restriction; never a care to stem the awful tide of heredity; never a reluctant purpose to bring unwilling, unknowing, and irresponsible victims of hereditary disease into a world where every environment spells destruction.

In no realm of human thought is the problem of keener interest or wider significance than in the domain of mental or nervous disease. Never, till my experience in asylum service, did I fully realize the truth of Sacred Writ, that the sins of the father shall be visited upon the children, to the third and fourth generation. Why this limitation? Because nature fortunately exhausts its atavism and degeneracy by the time the fourth generation is reached, and the tribe happily becomes extinct.

Quoting from Dr. Berkley: "I learn from Darwin that the families of drunkards do not descend beyond the fourth generation, and Morel declares the plan of decadence as follows: In the first generation there are moral depravity and alcoholic excesses. In the second, drunkenness and maniacal outbursts. In the third, melancholia, hypochondria, and impulsive ideas, particularly those of murder. In the fourth the imbecile and idiot appear, and the family becomes extinct."

Fever is but an expression of toxines in the blood and the consequent poisoning of the higher nervous centers, and the very height of temperature may prove a conservative process of nature which tends to destroy the invading micro-organisms and their destructive poisons. So insanity and high-grade nervous diseases constitute an unhealthy temperature of the body and mind, which, rising to its fullest height, reacts upon itself and destroys its own *"fons et origo."*

I would have you remember that insanity is not infrequently a convertible term in the process of heredity. It is a tree which will bear many different kinds of fruit upon its outspread branches. Dr. Peterson says, in determining the factor of heredity, "that we must not be content with ascertaining th e existence of psycnoses in the ascendants, but must seek by careful interrogation of various members of the family for some of the hereditary equivalents, such as epilepsy, chorea, hysteria, neurasthenia, somnambulism, migraine. Organic diseases of the central nervous system, criminal tendencies, eccentricities of character, drunkenness, etc., for these equivalents are interchangeable from one generation to another, and are simply evidences of instability of the nervous system."

Dr. Henry Maudsley states that "idiocy is indeed a manufactured article," and further that "multitudes of human beings come into the world with a destiny against which they have neither the will nor the power to contend; they are the stepchildren of nature, and groan under the worst of all tyrannies, *the tyranny of a bad organization.*"

It is safe to say that heredity plays its baneful rôle in at least

60 per cent of all cases of insanity, and, perhaps, the proportion of nervous diseases is higher still. Gender and age and nationality are relatively unimportant. The important factors in the production of insanity are heredity, alcoholism, in its various forms; the common grind and routine of life and its stress and strain. If these factors could be eliminated, and I believe much can be done in this direction, insanity will no longer remain a stalking spectre to every home.

Second—Vice and Crime. It may be said in this connection that criminals are not infrequently like poets and orators, born, not made.

I would not be among those who undermine social order and belittle statutory penalties by teaching anæmic ideas of criminal responsibility, but it can not be successfully denied that children are sometimes born with an hereditary tendency to lives of vice and crime, irresistible in their very nature. With good moral environments and proper education much may be done to overcome this, and some wayward one saved to social order and personal purity, but alas! in too many instances, moral perversion is but another name for hereditary mental depravity. Lives of disappointment and failure are but natural and inevitable results.

Just here I might call your attention to one of the disastrous results of physical disease of wide distribution among the children in early childhood. I refer to typhoid fever, which not infrequently results in cerebral hemorrhage, and consequent hemiphlegia, with arrested mental development and acquired imbecility, perhaps of a high order and not always recognized, especially if all traces of paralysis have disappeared, and where moral perversion may become marked in later life, and families greatly humiliated in ignorance of the fact that mental irresponsibility is the basis and bottom of the wrongful conduct in these unfortunate children.

Third—The Burden of Taxation. If the problem of insanity makes no other appeal to the public mind than its everlasting drain upon the pockets of our people, it becomes one to challenge serious consideration. Every man is willing to stop and count the cost of war, of armies, and navies, of education, of administering of justice, and the care of the criminal classes; why not due consideration to the expense of taking care of the mentally defective? Let me present a few figures: In 1861, when Texas had but one institution for the insane, with a population of fifty, the Legislature made an appropriation of $12,000 to cover the entire cost of maintenance of the insane population for the whole year, while last year,—only forty-four years later—the smallest and newest of our institutions, which had only been opened six months, expended nearly twice as much in current expenses, and had a total appropriation of $105,900, or nearly nine times as much, as was appropriated for the first year's maintenance of the original institution. Texas appropriated, for the fiscal year 1903-04 the large sum of $780,155 for the care of the insane. This is more than sixty-five times the amount of appropriations less than half a century ago, and we must remember that there are probably 500 lunatics still unprovided for in the State. They are those taken care of in private houses, county jails, and poor houses, and that, too, at a greater cost to taxpayers than State care.

Shall we heed these figures, which are destined to grow and multiply, till the lawmakers shall stand affrighted and people shall tremble at their burdens? In this country our ratio of insanity to the general population is about 1 to 500, while in older trans-oceanic countries the proportion has fallen to 1 to 100, and the end is not yet. What will be the burden of public taxation when Texas has expanded her population to cover her vast area and has admitted all sorts of alien and degenerate ancestry? The figures will stagger us, yet New York is smaller in area and probably has a smaller population than Texas will have in fifty years; and yet New York has more than 25,000 insane people, and expended, in 1904, $4,402,380.32 for their care. The cost of education is great, but it is an expenditure for the improvement of the race in body, mind, and morals, and no one will question its wisdom or necessity, and yet all this vast expense is more nearly approached than the public imagine, by the cost of taking care of the degenerate and defective, the criminals and the vicious, who perpetuate their kind. It is, moreover, an obligation that must be met; it appeals to a vital sense of the human heart, and its neglect would produce a moral wreck in the individual and the nation. Great characters in history have usually been those with the broadest sympathy and the most humane and tender feelings, and the world still prizes such characters and such ideals. It becomes us, then, to look well to the dispensation of our charity to the weak and helpless, for it must always be generous as well as just, and it behooves us, as wise husbandmen of our individual and national resources, and our destiny as a race, to study carefully this great social and economic problem, and devise some effective and humane means of reducing the baneful influences and evidences of insanity and its allied neuropsychoses and economize the cost in dollars, in lives, in homes, and in hearts.

